# Association of circulating MtDNA with CVD in hemodialysis patients and in vitro effect of exogenous MtDNA on cardiac microvascular inflammation

**DOI:** 10.1186/s12872-023-03104-2

**Published:** 2023-02-08

**Authors:** Zhen Fan, Ya Feng, Li Zang, Yi Guo, Xiao-yi Zhong

**Affiliations:** 1 Department of Geriatrics, Sichuan Provincial People’s Hospital, University of Electronic Science and Technology of China, Chengdu, Sichuan China; 2 Department of Nephrology, Clinical Medical College, The First Affiliated Hospital of Chengdu Medical College, Chengdu, Sichuan China; 3 Department of Neurology, Sichuan Provincial People’s Hospital, University of Electronic Science and Technology of China, No. 32, West Section 2, Yihuan Road, Qingyang District, Chengdu, 610072 Sichuan China; 4 Department of Nephrology, Xinqiao Hospital, Army Medical University (Third Military Medical University), No. 83 Xinqiao Zhengjie, Shapingba District, Chongqing, 400037 China

**Keywords:** Maintenance hemodialysis, Cardiovascular disease, Cardiac microvascular endothelial cells, Mitochondrial DNA, Inflammation

## Abstract

**Background:**

Chronic kidney disease (CKD) patients sustain a fairly high prevalence of cardiovascular disease (CVD). Microvascular inflammation is an early manifestation of CVD, and the released mitochondrial DNA (MtDNA) has been proposed to be a crucial integrator of inflammatory signals. Herein, the aim of this study was to determine the relationship between CVD, microvessel, and circulating MtDNA in the settings of uremia.

**Methods:**

Forty-two maintenance hemodialysis (MHD) patients and 36 health controls were enrolled in this study. Plasma cell-free MtDNA was detected by TaqMan-based qPCR assay. CVD risk markers including high-sensitive C-reactive protein (Hs-CRP), monocyte chemoattractant protein-1 (MCP-1), fibrinogen, and erythrocyte sedimentation rate (ESR) were measured by standard assays. Ten-year CVD risk was calculated from the Framingham risk score (FRS) model. In vitro study, human cardiac microvascular endothelial cells (HCMECs) were incubated with normal or uremic serum, with or without exogenous MtDNA. Intracellular toll-like receptor 9 (TLR9), adhesion molecule 1 (ICAM-1), MCP-1 and tumor necrosis factor-α (TNF-α) and cytosolic MtDNA were detected by qPCR.

**Results:**

Plasma MtDNA in MHD patients was significantly higher than healthy controls (4.74 vs. 2.41 × 10^5^ copies/mL; *p* = 0.000). Subsequently, the MHD patients were classified into two groups based on the MtDNA median (4.34 × 10^5^ copies/mL). In stratified analyses, the levels of Hs-CRP (5.02 vs. 3.73 mg/L; *p* = 0.042) and MCP-l (99.97 vs. 64.72 pg/mL; *p* = 0.008) and FRS (21.80 vs. 16.52; *p* = 0.016) in the high plasma MtDNA group were higher than those in the low plasma MtDNA group. In vitro study, we found that exogenous MtDNA aggravated uremic serum-induced microvascular inflammation (ICAM-1 and TNF-α) in HCMECs (all *p* < 0.05). Besides, the addition of MtDNA to the medium resulted in a further increase in cytosolic MtDNA and TLR9 levels in uremic serum-treated cells (all *p* < 0.05). In patients with MHD, MtDNA levels in plasma were significantly reduced after a single routine hemodialysis (pre 4.47 vs. post 3.45 × 10^5^ copies/mL; *p* = 0.001) or hemodiafiltration (pre 4.85 vs. post 4.09 × 10^5^ copies/mL; *p* = 0.001). These two approaches seem similar in terms of MtDNA clearance rate (21.26% vs. 11.94%; *p* = 0.172).

**Conclusions:**

Overall, the present study suggests that MtDNA released into the circulation under the uremic toxin environment may adversely affect the cardiovascular system by exacerbating microvascular inflammation, and that reducing circulating MtDNA might be a future therapeutic strategy for the prevention of MHD-related CVD.

## Background

Cardiovascular disease (CVD) frequently occurs in chronic kidney disease (CKD) patients, especially those undergoing maintenance hemodialysis (MHD) therapy [1]. However, traditional risk factors, including hypertension, diabetes and hypercholesterolemia, only account partially for the CVD burden in CKD [2]. CKD-specific mechanisms explaining the link between CKD and CVD are still incompletely understood. Patients with CKD often manifest endothelial dysfunction [3]. Endothelial cell (EC) dysfunction leads to the disintegration of macrovascular and microvascular structures and contributes to CVD [4]. Therefore, it is imperative to investigate how CKD contributes to endothelial damage and dysfunction.

Mounting evidence suggests that the earliest presymptomatic functional and pathological changes occur at the level of microcirculation in hearts [5]. The cardiac microvasculature primarily consists of cardiac microvascular endothelial cells (CMECs) [6] which are both active participants and regulators of inflammatory processes [7]. During the transition from acute to chronic inflammation or from innate to adaptive immunity, CMECs characteristics change through CMECs activation, recruitment of inflammatory cells, and increased vascular permeability. These pathological changes eventually lead to endothelial dysfunction and remodeling [7, 8]. Hence, identification of the inflammatory target is critical to treat the ‘inflammation’ component of this disease.

Mitochondria are cytoplasmic organelles within eukaryotic cells that carry their own genomic materials apart from those in the nucleus. The endosymbiotic theory suggests that mitochondria are derived from a formerly free-living α-proteobacterium [9]. Owing to its bacterial origins, the extracellular release of mitochondrial components—in particular, cell-free mitochondrial DNA (MtDNA), during stress responses or cell death can trigger an immune response through activation of pathogen recognition receptors (PPRs) [10–12]. In corroboration with other studies [13, 14], we previously reported increased levels of free-circulating MtDNA and altered mitochondrial homeostasis in MHD patients [15]. Whereas, the association of circulating MtDNA with CKD-related CVD and its potential contribution to CMEC inflammation has never been established.

The aim of the present study was to investigate the association between circulating MtDNA and CVD risk in MHD patients, as well as its effect on CMEC inflammation in vitro. Additionally, MtDNA levels were measured before and after hemodialysis treatment to assess the effectiveness of different dialysis modalities on its removal.

## Materials and methods

### Participant eligibility

From November 2021 to May 2022, MHD patients from the Center of Kidney Disease of First Affiliated Hospital of Chengdu Medical College were recruited. MHD patients were defined as follows: (a) age above 40; and (b) patients undergoing regular hemodialysis prescription (3 times per week) for at least 6 months. The age‐ and sex‐matched healthy controls (HC) included individuals who attended routine health exams at the same hospital; these patients had no history or clinical evidence of any renal diseases. The exclusion criteria for all subjects were as follows: (a) active inflammatory diseases within the last 3 months; (b) malignant tumors; (c) immune system diseases; (d) active liver disease; and (e) acute cardiovascular and cerebrovascular disease.

### Clinical study design

A total of 42 MHD patients and 36 control participants took part in this case-control cross-sectional study and, among them, 32 MHD patients agreed to participate in the next prospective cohort study. In the prospective part of the study, MHD patients were randomly and equally divided into two groups according to the dialysis patterns: hemodialysis (HD, *n* = 16) with low flux synthetic filters; hemodiafiltration (HDF, *n* = 16) with high flux polysulfone filter. Each hemodialysis treatment lasted for 4 h using a dialyzer with a blood flow rate of 200–250 mL/min and a dialysate flow of 500 mL/min. HDF was conducted using a post-dilution replacement fluid with a volume of 30% of the ultrafiltration blood flow. The study was performed according to the Declaration of Helsinki and approved by the ethics committee of First Affiliated Hospital of Chengdu Medical College (ethical approval number: 2021CYFYIRB-BA-61-01). Written informed consent was obtained from all patients.

### General data collection

General information was collected on each participant including age, sex, dialysis vintage, previous disease history (hypertension, diabetes, hyperlipemia and coronary heart disease), and personal history (smoking and alcohol consumption).

### Laboratory measurements

For dialysis participants, blood samples were collected prior to and following the dialysis sessions. For control, peripheral venous blood samples were collected from healthy individuals. Routine laboratory parameters were measured directly after blood withdrawal by standardized methods for total cholesterol (TC), high-density lipoprotein cholesterol (HDL-C), low-density lipoprotein cholesterol (LDL-C), glycosylated hemoglobin (GH), blood urea nitrogen (BUN), serum creatinine (SCr), high-sensitive C-reactive protein (Hs-CRP), fibrinogen and erythrocyte sedimentation rate (ESR) in clinical laboratories of the participating hospitals. Monocyte chemoattractant protein-1 (MCP-1) was measured by ELISA (Byotimes, China). Routinely measured equilibrated Kt/V values (Daugirdas equation [16]) were extracted from the patients’ medical records.

### Measurement of MtDNA in human plasma

DNA from plasma was extracted using the DNA Blood Mini Kit (Qiagen, USA). A Nanodrop spectrophotometer was utilized for quantifying extracted DNA. MtDNA-encoded cytochrome B gene was amplified by TaqMan qPCR. The PCR primers and TaqMan probes were designed and synthesized by TsingKe Biological Technology (TsingKe, China). Cytochrome B: sense primer, 5′‐CGCTACCTTCACGCCAATG‐3′, antisense primer, 5′‐CGATGTGTAGGAAGAGGCAGATAA‐3′, FAM‐labeled TAMRA quenched probes, 5′‐CGCCTCAATATTC‐3′. The linearity of the quantitative assay was assessed using the template cloned into plasmid DNA and serially diluted to prepare a series of calibrators with known concentrations. Quantification of MtDNA was accomplished based on this standard curve by consulting a prior method [17]. Results were presented as MtDNA × 10^5^ copies per mL.

### Framingham risk score calculation

Framingham risk score (FRS) was calculated for men and women to estimate the 10-year risk of a CVD event using variables including age, sex, total cholesterol, high-density lipoprotein cholesterol, presence or absence of smoking, presence or absence of diabetes, systolic blood pressure, and status of blood pressure medications.

### Experimental cell culture and treatments

Human cardiac microvascular endothelial cells (HCMECs) purchased from Procell (China) were maintained in endothelial cell media (Procell, China) with 20% fetal bovine serum (FBS) and 1% penicillin plus streptomycin. Afterwards, the standard medium (20% FBS) was exchanged with medium containing 20% normal or uremic human serum with or without MtDNA (20 µg/mL) for 24 h. All cells were cultured at 37 °C in a 5% CO^2^ incubator.

### Serum pool from uremic patients and healthy volunteers

Uremic serum was collected from 20 hemodialysis patients prior to a regularly scheduled hemodialysis session. Serum drawn from 20 healthy donors served as normal controls. Serum from all individuals was pooled, filtered, and aliquoted in small vials to be frozen at – 80 °C for cell experiments.

### Isolation of mitochondria and MtDNA preparation

Mitochondria-enriched fractions were isolated from HCMECs with a Mitochondrial Isolation Kit (Byotimes, China). Total MtDNA was extracted from the isolated mitochondria under sterile conditions using the Mitochondria DNA Isolation kit (Abcam, USA). The concentration of MtDNA was determined by Nanodrop spectrophotometer.

### RNA isolation, cDNA preparation and qPCR

Total RNA from HCMECs was extracted using TRIzol Reagent (Sangong, China). Then, RNA was reverse transcribed into the cDNA using a reverse-transcribed (RT) reagent (Takara, Japan). After RT reaction, 2 µL of cDNA was used per qPCR reaction. PCR was performed using SYBR Green Premix (Takara, Japan). Gene expression of intracellular adhesion molecule-1 (ICAM-1), MCP-1, tumor necrosis factor-α (TNF-α) and toll-like receptor 9 (TLR9) was normalized to glyceraldehyde 3-phosphate dehydrogenase (GAPDH) and compared with control group. The data were analyzed by delta-delta Ct method. The primers were as follows: ICAM-1: 5′–ATCAAATGGGGCTGGGA–3′ (forward) and 5′–GGGGAAGGGAGGAATAAGG–3′ (reverse); MCP-1: 5′–CAAGCAGAAGTGGGTTCAG–3′ (forward) and 5′–GGGAAAGCTAGGGGAAAA–3′ (reverse); TNF-α: 5′–GGAAAGGACACCATGAGC–3′ and 5′–CCACGATCAGGAAGGAGA–3′ (reverse); TLR9: 5′–TGAAGGAGCTGGACATGC–3′ (forward) and 5′–GGGCCTGGTTGATGAAGT–3′ (reverse); GAPDH: 5′–GGGGCTCTCCAGAACATC–3′ (forward) and 5′–TGACACGTTGGCAGTGG–3′ (reverse).

### Measurement of MtDNA in the cytosol

Mitochondrial isolation kits (Byotimes, China) were utilized for preparing mitochondrion-free cytosolic fractions. DNA Blood Mini Kit (QIAamp, USA) was applied for acquiring cytosolic DNA. qPCR was performed to detect mitochondrial cytochrome B. The qPCR-based assessment of nuclear DNA (nDNA) was achieved with primers hybridized to 18S rDNA sequences (encoding 18S rRNA). Next step was normalization of the MtDNA expression against those of nDNA. The primers were as follows: cytochrome B: 5′–TTCTTGCACGAAACGGGATC–3′ (forward) and 5′–AAGCCGAGGGCGTCTTTGATTGTGT–3′ (reverse); 18S rDNA: 5′–GGAGTATGGTTGCAAAGCTGA–3′ (forward) and 5′–ATCTGTCAATCCTGTCCGTGT–3′ (reverse).

### Statistical analyses

Data are expressed as means ± standard deviation (SD) or medium (25th and 75th percentiles) for continuous variables. The distribution of the data was tested using the Kolmogorov–Smirnov test. Normally distributed data were compared by the paired or unpaired *t*-test as appropriate. Nonnormally distributed data were analyzed by either a Wilcoxon signed-rank test for paired samples or a Mann-Whitney U test for unpaired samples. Categorical data between two groups were compared using the chi-square test (χ^2^ test) with Fisher’s exact test. Values of *p* < 0.05 were considered statistically significant. All statistical analyses were conducted using SPSS 18.0.

## Results

### MHD subjects exhibit higher levels of plasma MtDNA

A total of 78 subjects (42 hemodialysis patients and 36 age- and sex-matched healthy controls) were recruited in the current study. Demographic, clinical, and laboratory characteristics of participants were presented in Table 1. No significant differences were found between cases and controls with respect to gender and age distributions. As expected, hemodialysis patients had a higher prevalence of diabetes and higher levels of GH, BUN and SCr than controls. In the health controls, plasma MtDNA could be detected at a low concentration of 2.41 × 10^5^ copies/mL, while it was higher in hemodialysis patients before hemodialysis initiation, with a concentration of 4.74 × 10^5^ copies/mL.

**Table 1:** MHD subjects exhibit higher levels of plasma MtDNA

Variable	Healthy	Hemodialysis	*t*/χ^2^	*p*
Subject number	36	42		
Age (years)	60.77 ± 8.98	59.40 ± 9.29	0.661	0.511
Male, n (%)	18 (50.0%)	25 (59.5%)	0.711	0.399
Dialysis vintage (years)	NS	3.76 ± 1.64		
Hypertension, n (%)	8 (22.2%)	13 (30.9%)	0.751	0.386
Diabetes, n (%)	6 (16.7%)	16 (38.1%)	4.396	0.036
Hyperlipemia, n (%)	17 (47.2%)	15 (35.7%)	1.061	0.303
CHD, n (%)	2 (5.6%)	7 (16.7%)	2.345	0.126
Smoking, n (%)	6 (16.7%)	9 (21.4%)	0.283	0.595
Alcohol, n (%)	12 (33.3%)	10 (23.8%)	0.869	0.351
TC (mmol/L)	4.14 ± 0.93	4.49 ± 1.22	− 1.378	0.172
HDL-C (mmol/L)	1.24 ± 0.36	1.14 ± 0.28	1.338	0.185
LDL-C (mmol/L)	2.48 ± 1.09	2.38 ± 1.01	0.423	0.674
GH (%)	5.94 ± 1.13	6.69 ± 1.28	− 2.703	0.008
BUN (mmol/L)	5.89 ± 1.72	23.99 ± 5.75	− 18.163	0.000
SCr (umol/L)	68.01 ± 20.67	951.54 ± 177.05	− 29.741	0.000
Plasma MtDNA (10^5^ copies/mL)	2.41 ± 1.49	4.74 ± 1.85	− 6.043	0.000

CHD: Coronary heart disease; TC: total cholesterol; HDL-C: high-density lipoprotein cholesterol; LDL-C: low-density lipoprotein cholesterol; GH: glycosylated hemoglobin; BUN: blood urea nitrogen; SCr: serum creatinine; MtDNA: mitochondrial DNA

### Plasma MtDNA is linked to CVD risk in MHD patients

Subgroup analyses were subsequently performed according to the median value of plasma MtDNA in MHD patients (low, < 4.34 × 10^5^ copies/mL, *n* = 21; high, ≥ 4.34 × 10^5^ copies/mL, *n* = 21). CVD risk markers were compared between the subgroups and presented in Table 2. The results showed that serum levels of Hs-CRP and MCP-l, and FRS in high plasma MtDNA group were significantly higher than those in low plasma MtDNA group. Our findings link high plasma MtDNA to elevations in cardiovascular risk markers that are established to be strong predictors of future cardiovascular events. However, these data raise the question as to whether these changes may be causally related.

**Table 2:** Plasma MtDNA is linked to CVD risk in MHD patients

Variable	Low plasma MtDNA	High plasma MtDNA	t	*p*
Subject number	21	21		
Hs-CRP (mg/L)	3.73 ± 1.89	5.02 ± 2.06	− 2.104	0.042
MCP-1 (pg/mL)	64.72 ± 42.27	99.97 ± 39.63	− 2.788	0.008
Fibrinogen (g/L)	2.61 ± 0.32	2.56 ± 0.35	0.476	0.637
ESR (mm/hour)	17.26 ± 6.54	19.37 ± 6.92	− 1.012	0.317
FRS	16.52 ± 6.39	21.80 ± 7.18	− 2.520	0.016

MtDNA: Mitochondrial DNA; Hs-CRP: high-sensitive C-reactive protein; MCP-1: monocyte chemoattractant protein-1; ESR: erythrocyte sedimentation rate; FRS: framingham risk score

### Exogenous MtDNA aggravates uremic serum-induced cardiac microvascular inflammation in HCMECs

**Fig. 1:**
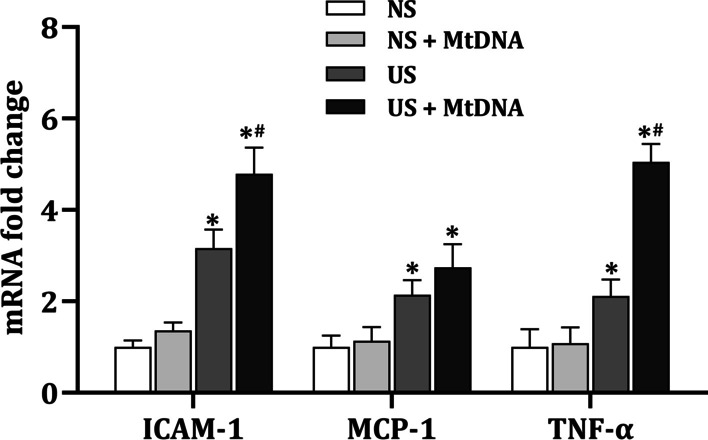
Exogenous MtDNA aggravates uremic serum-induced microvascular inflammation in HCMECs. PCR analysis was performed on ICAM-1, MCP-1 and TNF-α, in which GAPDH was taken as internal reference. Each experiment was performed in triplicate and repeated for three times. *p < 0.05 compared to normal serum group; # *p* < 0.05 compared to uremic serum group. Data are mean ± SD. NS, normal serum; US, uremic serum

Microvascular inflammation is increasingly recognized as an important component of CVD. To further explore the role of circulating MtDNA in CKD-related CVD, exogenous MtDNA was added to HCMECs either in the presence or absence of uremic serum, and the changes of endothelial inflammation-related cytokines were analyzed by qPCR. The qPCR examination showed a remarkable increase in mRNA level of ICAM-1, MCP-1 and TNF-α after uremic serum treatment. Interestingly, although MtDNA alone did not cause any significant changes to cytokine expression, exogenous MtDNA aggravated uremic serum-induced upregulation of ICAM-1 and TNF-α (Fig. 1). These results demonstrate that extracellular MtDNA are able to induce cardiac microvascular inflammation in the uremic milieu.

### HCMECs internalize extracellular MtDNA in the uremic milieu

**Fig. 2:**
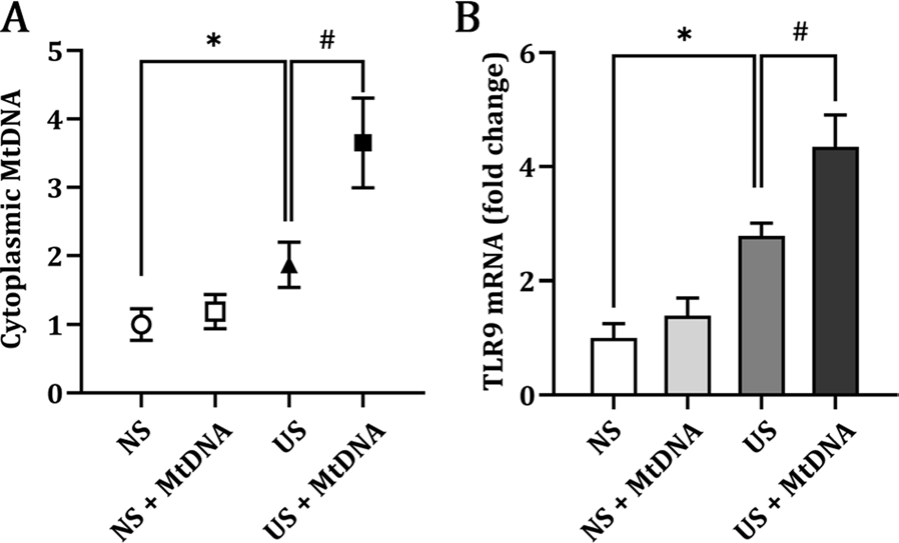
HCMECs internalize extracellular MtDNA in the uremic milieu. **A** PCR analysis of cytochrome B abundance in cytosolic fractions. The internal reference was 18S rDNA. **B** PCR analysis was performed on TLR9, in which GAPDH was taken as internal reference. Each experiment was performed in triplicate and repeated for three times. *p < 0.05 compared to normal serum group; #*p* < 0.05 compared to uremic serum group. Data are mean ± SD. NS, normal serum; US, uremic serum

MtDNA, similar to bacterial DNA and rich in unmethylated CpG motif, are immunostimulatory through its putative receptor, TLR9. It is proposed that TLR9 is initially localized in the endoplasmic reticulum and translocates to endolysosomes upon stimulation with CpG ligands [18]. Thus, extracellular MtDNA must re-enter the cell and bind its intracellular receptor to function. As shown in Fig. 2A, the amount of cytosolic MtDNA was significantly higher in cells treated with uremic serum than in the control cells. Treatment with MtDNA alone had no obvious effect on cytosolic MtDNA. However, in the presence of uremic serum, the addition of MtDNA to the medium resulted in a 3.6-fold increase in cytosolic MtDNA in HCMECs, which was 1.9-fold higher than in the uremic serum alone group. A similar tendency was also observed in intracellular TLR9 mRNA expression (Fig. 2B). Overall, these data confirmed that exogenous MtDNA can indeed be internalized by HCMECs under uremic environment.

### The effectiveness of different dialysis modalities on MtDNA removal

**Fig. 3:**
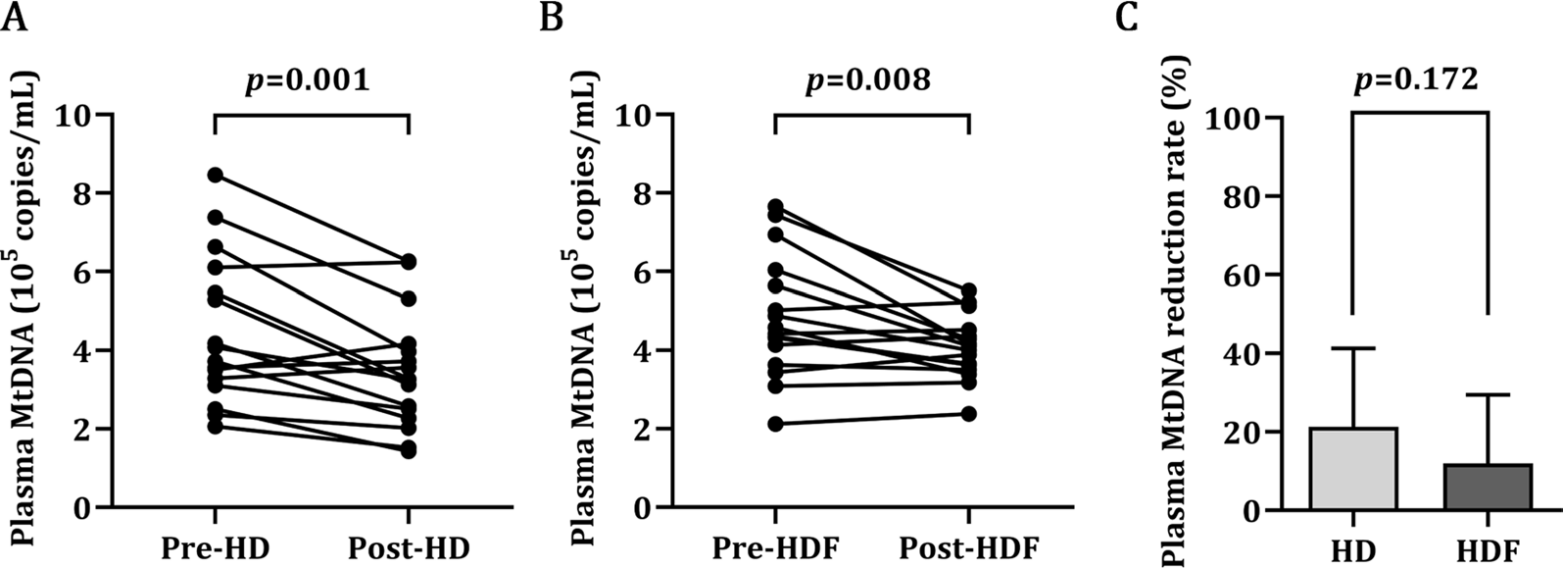
The effectiveness of different dialysis modalities on MtDNA removal. **A** The effectiveness of HD on plasma MtDNA (*n* = 16). **B** The effectiveness of HDF on plasma MtDNA (*n* = 16). **C** Comparison of plasma MtDNA reduction rate between the two groups. HD, hemodialysis; HDF, hemodiafiltration

A total of 42 hemodialysis patients were screened, 10 patients declined to participate, and the remaining 32 patients were included for this prospective cohort study. The patients were randomly divided into two groups: HD, *n* = 16 and HDF, *n* = 16. The abundance of plasma MtDNA before and after a single dialysis was evaluated. At baseline, no significant differences were observed between the two groups regarding clinical and biological variables, including gender, age, dialysis vintage, Kt/V, BUN, Scr and pre-treatment plasma MtDNA (Table 3). In the patients undergoing HD, plasma MtDNA was decreased from 4.47 × 10^5^ copies/mL (pre‑treatment) to 3.45 × 10^5^ copies/mL (post‑treatment), the difference was identified to be statistically significant (Fig. 3A). Meanwhile, the HDF group also demonstrated a significantly decreased plasma MtDNA from 4.85 × 10^5^ copies/mL to 4.09 × 10^5^ copies/mL (Fig. 3B). Nevertheless, there was no significant difference in the reduction rate of plasma MtDNA between the two blood purification methods (Fig. 3C), which suggested that the efficacy of the two MtDNA clearance methods was similar.

**Table 3:** Clinical and biological variables of the two groups prior to treatment

Variable	HD	HDF	*t*/χ^2^	*p*
Subject number	16	16		
Males, n (%)	11 (68.8%)	9 (56.3%)	0.533	0.465
Age (years)	60.37 ± 9.25	59.06 ± 9.82	0.389	0.700
Dialysis vintage (years)	4.18 ± 1.64	3.43 ± 1.63	1.296	0.205
Kt/V	1.45 ± 0.11	1.43 ± 0.10	0.418	0.679
BUN (mmol/L)	24.76 ± 6.36	24.24 ± 5.19	0.252	0.803
SCr (µmol/L)	964.83 ± 161.13	942.25 ± 181.24	0.584	0.712
Pre-treatment plasma MtDNA (10^5^ copies/mL)	4.47 ± 1.88	4.85 ± 1.55	− 0.618	0.542

HD: Hemodialysis; HDF: hemodiafiltration; Kt/V: formula for dialysis efficiency; BUN: blood urea nitrogen; SCr: serum creatinine; MtDNA: mitochondrial DNA

## Discussion

CKD patients, especially those receiving MHD, sustain a fairly high prevalence of CVD. Cardiac microvascular inflammation is a key step in the initiation, development and progression of a series of CVD [19]. Recently, released MtDNA has been proposed to be a crucial integrator of inflammatory signals. Given this, it would be of interest to elucidate the relationship among circulating MtDNA, microvascular inflammation and CVD in the CKD population. The primary findings of our study were as follows. (a) Circulating MtDNA level is significantly higher in MHD patients than in healthy participants, and higher circulating MtDNA is associated with greater risks for CVD events. (b) Exogenous MtDNA aggravates uremic serum-induced microvascular inflammation in HCMECs. (c) Extracellular MtDNA can be internalized by HCMECs, which perhaps leads to the activation of downstream signaling pathways that enhance inflammatory response. (d) Both low flux-HD and HDF can partially reduce the circulating MtDNA.

Mitochondria are the only other subcellular structures with their own DNA; each cell has multiple mitochondria, and each mitochondria contain several copies of MtDNA. MtDNA are usually released by cells undergoing stress or having pathological events. Unlike genomic DNA, MtDNA contains hypomethylated CpG motifs similar to bacterial DNA. Thus, the extracellular MtDNA may act as a damage-associated molecular pattern (DAMP) in promoting sterile inflammation [20]. Currently, this so-called DAMP has been found be increased in CVD (such as heart failure, atherosclerosis and coronary syndromes) and associated risk conditions (including arterial hypertension, diabetes mellitus, and hypercholesterolemia), and is considered to be a predictor of adverse clinical outcomes [21]. Whether these effects are also present in patients with MHD remain to be made clear. Consistent with previous studies [13, 14], our data showed that the level of MtDNA in plasma was higher in MHD patients than in healthy controls. In stratified analysis, we observed a high consistency in plasma MtDNA with CVD risk markers. However, correlations do not prove causality. To establish a cause-effect relationship, it is important to interpret how circulating MtDNA contributes to CVD initiation and progression.

Endothelial dysfunction is considered as the earliest stage in the development of cardiovascular disease, prior to the clinical manifestation of symptoms [22]. Apparently, endothelial dysfunction occurs in the microcirculation before affecting macrovascular structures [23, 24], but most research in CKD-related CVD has been devoted to macrovascular complications [25]. CMECs, which account for one-third of the total number of heart cells, is the major cell type in the processes of cardiovascular angiogenesis and play a critical role in maintaining sufficient cardiac perfusion. In this regard, understanding the intracellular mechanism that regulates uremia-induced injury in CMECs is important for reconditioning the injured endothelium. Available evidence indicates that endothelial dysfunction is often closely related to a state of inflammation [26]. In our research, no inflammatory response was observed to MtDNA treatment alone. However, in the presence of uremic serum, the addition of MtDNA aggravated uremia-induced microvascular inflammation. Exogenous MtDNA also resulted in a further increase in cytosolic MtDNA and TLR9 levels in uremia-conditioned HCMECs. Self-double-stranded DNA is generally nuclear, whereas TLR9 recognizes cytosolic foreign DNA (such as MtDNA) in intracellular endosomes [27]. Thus, these data suggested that the extracellular MtDNA could be effectively internalized by CMECs, although the exact cell entry mechanisms have not been deciphered yet. In our study, exposure to uremic serum is sufficient to cause an increase in cytosolic MtDNA. Nevertheless, it should be noted that MtDNA can be released from CMECs’ own mitochondria. As previously reported, the number of mitochondria is modest in endothelial cells compared to other cell types with a higher energy requirement [28]; for example, in rats, endothelial mitochondria compose 2–6% of the cell volume, as opposed to 32% in cardiac myocytes [26]. Based on these studies, we speculated that uremia-induced increases in cytosolic MtDNA in CMECs may be primarily due to the MtDNA influx from the extracellular space. Many types of cells are able to respond to MtDNA outside cells. Immunocytes, for instance, are capable of identifying, internalizing and generating an immunoresponse following extracellular MtDNA activation [29]. Besides, cellular dysfunction and sterile injury secondary to exogenous MtDNA can also be found in lung epithelial cell, cardiomyocyte, and vascular smooth muscle cell [30–32]. To our knowledge, this is the first report demonstrated a pro-inflammatory activity for extracellular MtDNA in CMECs under the uremic condition.

Hemodialysis or blood filtration, to remove toxins is the primary method of treating ESRD patients. We found that the two blood purification methods were effective in the clearance of plasma MtDNA; however, there were no statistically significant differences identified between two methods regarding the MtDNA reduction rate. In slight disagreement with our study, a previous finding suggested that HDF but not low-flux HD could partially reduce the level of plasma MtDNA [14]. But their vitro experiments showed that MtDNA could pass through the dialysis membranes not only in HDF but also in low-throughput HD [14]. It is generally accepted that HDF is more efficient in the removal of larger molecules. Since the removal of small solutes occurs primarily by diffusion, additional convection in HDF modality does not affect diffusive transport [33]. From these reasons, we considered that the form of MtDNA in plasma might be small molecular fragments and that increasing the frequency of routine HD beyond three times per week and performing longer dialysis sessions may improve the clearance of circulating MtDNA.

Our study has several limitations. First, the mechanism of how these exogenous MtDNAs enter the CMECs has not been elucidated. Second, whether exogenous MtDNA induces cardiac microvascular inflammation primarily through TLR9-dependent pathway remains to be determined. Third, diabetes mellitus is one of the main causes of CKD. In our study, a significant difference was observed in diabetes prevalence between the study groups. Therefore, it is difficult to rule out the effect of diabetes on circulating MtDNA in this cross-sectional study. Last, whether reducing circulating MtDNA can decrease the risk of CVD in dialysis patients will require validation in large prospective cohort studies.

## Conclusion

**Fig. 4:**
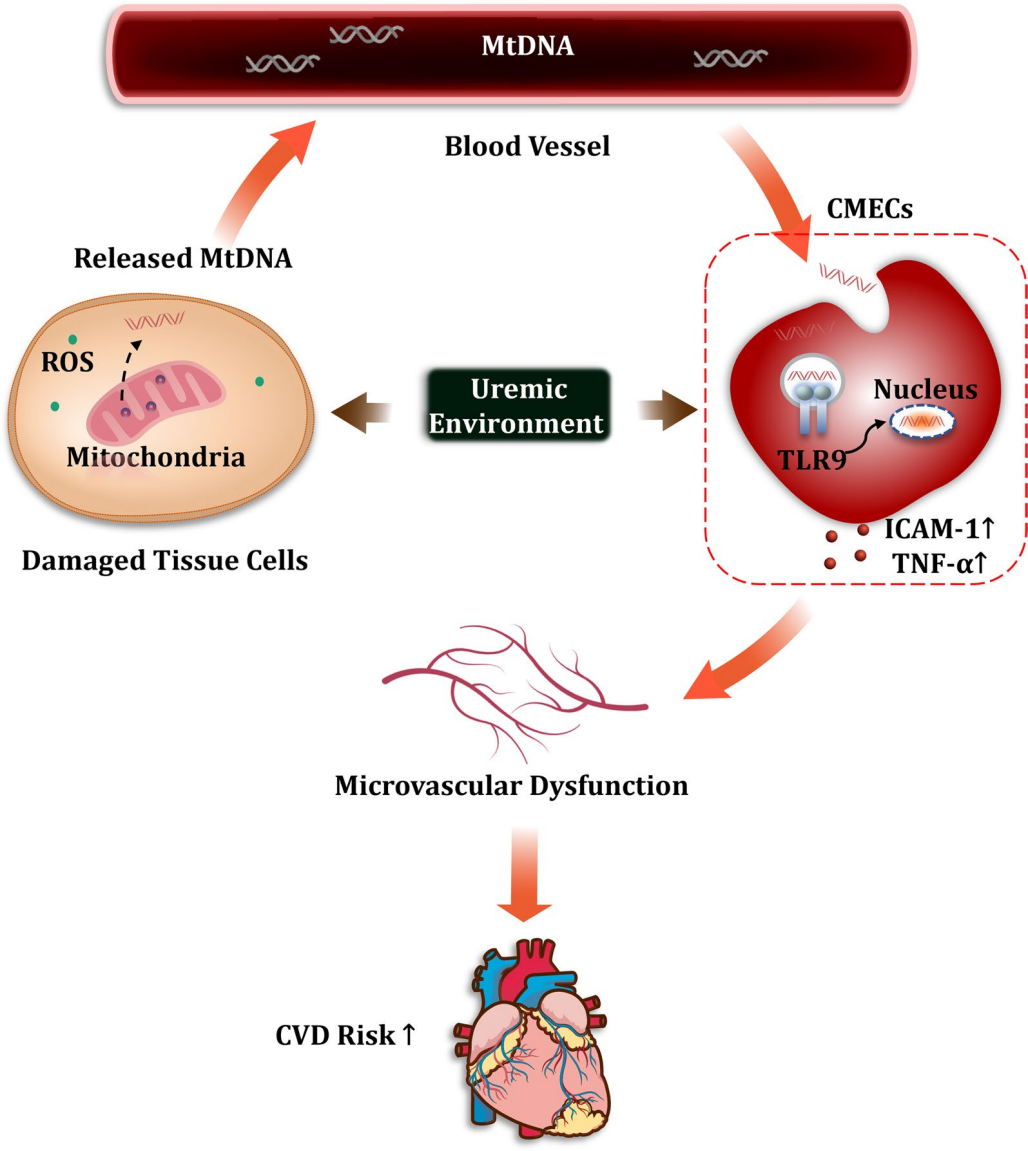
A schematic representation of MtDNA generation and diffusion and its role in CVD risk. Exposure to uremic milieu leads to redox imbalance and oxidative stress, which disrupts mitochondrial ultrastructure, causing the release of MtDNA into the cytosol and bloodstream. Free MtDNA molecules can be internalized by CMECs to elicit a sterile inflammatory response. This inflammatory challenge may subsequently lead to microvascular dysfunction and ultimately enhance CVD development

In summary, our study provides evidence highlighting the role of circulating MtDNA in MHD-related CVD. This study showed that higher circulating MtDNA was found in uremic serum. Additionally, exogenous MtDNA was able to induce cardiac microvascular inflammation in the uremic milieu. A schematic diagram of the proposed pathway is shown in Fig. 4. Altogether, this work supported that circulating MtDNA was considered as a hopeful therapeutic target for the treatment of MHD-related CVD. Future studies are still needed to confirm these findings.

## Data Availability

The datasets used and analyzed during the current study are available from the corresponding author on reasonable request.

## References

[1] Thompson S, James M, Wiebe N, Hemmelgarn B, Manns B, Klarenbach S, Tonelli M (2015). Cause of death in patients with Reduced Kidney Function. J Am Soc Nephrol.

[2] Matsushita K, Coresh J, Sang Y, Chalmers J, Fox C, Guallar E, Jafar T, Jassal SK, Landman GW, Muntner P (2015). Estimated glomerular filtration rate and albuminuria for prediction of cardiovascular outcomes: a collaborative meta-analysis of individual participant data. Lancet Diabetes Endocrinol.

[3] Fliser D, Wiecek A, Suleymanlar G, Ortiz A, Massy Z, Lindholm B, Martinez-Castelao A, Agarwal R, Jager KJ, Dekker FW (2011). The dysfunctional endothelium in CKD and in cardiovascular disease: mapping the origin(s) of cardiovascular problems in CKD and of kidney disease in cardiovascular conditions for a research agenda. Kidney Int Suppl.

[4] Benjamin EJ, Muntner P, Alonso A, Bittencourt MS, Callaway CW, Carson AP, Chamberlain AM, Chang AR, Cheng S, Das SR (2019). Heart disease and stroke statistics-2019 update: a report from the American Heart Association. Circulation.

[5] Bonetti PO, Lerman LO, Lerman A (2003). Endothelial dysfunction: a marker of atherosclerotic risk. Arterioscler Thromb Vasc Biol.

[6] Zhou H, Wang S, Zhu P, Hu S, Chen Y, Ren J (2018). Empagliflozin rescues diabetic myocardial microvascular injury via AMPK-mediated inhibition of mitochondrial fission. Redox Biol.

[7] Bai B, Yang Y, Wang Q, Li M, Tian C, Liu Y, Aung LHH, Li PF, Yu T, Chu XM (2020). NLRP3 inflammasome in endothelial dysfunction. Cell Death Dis.

[8] Pober JS, Sessa WC (2007). Evolving functions of endothelial cells in inflammation. Nat Rev Immunol.

[9] Itsara LS, Kennedy SR, Fox EJ, Yu S, Hewitt JJ, Sanchez-Contreras M, Cardozo-Pelaez F, Pallanck LJ (2014). Oxidative stress is not a major contributor to somatic mitochondrial DNA mutations. PLoS Genet.

[10] Rodríguez-Nuevo A, Díaz-Ramos A, Noguera E, Díaz-Sáez F, Duran X, Muñoz JP, Romero M, Plana N, Sebastián D, Tezze C (2018). Mitochondrial DNA and TLR9 drive muscle inflammation upon Opa1 deficiency. EMBO J.

[11] Bode C, Fox M, Tewary P, Steinhagen A, Ellerkmann RK, Klinman D, Baumgarten G, Hornung V, Steinhagen F (2016). Human plasmacytoid dentritic cells elicit a Type I Interferon response by sensing DNA via the cGAS-STING signaling pathway. Eur J Immunol.

[12] Zhong Z, Liang S, Sanchez-Lopez E, He F, Shalapour S, Lin XJ, Wong J, Ding S, Seki E, Schnabl B (2018). New mitochondrial DNA synthesis enables NLRP3 inflammasome activation. Nature.

[13] Zhang Y, Zhao Y, Wen S, Yan R, Yang Q, Chen H (2017). Associations of mitochondrial haplogroups and mitochondrial DNA copy numbers with end-stage renal disease in a Han population. Mitochondrial DNA Part A DNA Mapp Seq Anal.

[14] Cao H, Ye H, Sun Z, Shen X, Song Z, Wu X, He W, Dai C, Yang J (2014). Circulatory mitochondrial DNA is a pro-inflammatory agent in maintenance hemodialysis patients. PLOS ONE.

[15] Zhong XY, Guo Y, Fan Z (2022). Increased level of free-circulating MtDNA in maintenance hemodialysis patients: possible role in systemic inflammation. J Clin Lab Anal.

[16] Daugirdas JT (1993). Second generation logarithmic estimates of single-pool variable volume Kt/V: an analysis of error. J Am Soc Nephrol.

[17] Chiu RW, Chan LY, Lam NY, Tsui NB, Ng EK, Rainer TH, Lo YM (2003). Quantitative analysis of circulating mitochondrial DNA in plasma. Clin Chem.

[18] Hemmi H, Takeuchi O, Kawai T, Kaisho T, Sato S, Sanjo H, Matsumoto M, Hoshino K, Wagner H, Takeda K (2000). A Toll-like receptor recognizes bacterial DNA. Nature.

[19] Cuijpers I, Simmonds SJ, van Bilsen M, Czarnowska E, González Miqueo A, Heymans S, Kuhn AR, Mulder P, Ratajska A, Jones EAV (2020). Microvascular and lymphatic dysfunction in HFpEF and its associated comorbidities. Basic Res Cardiol.

[20] West AP, Shadel GS (2017). Mitochondrial DNA in innate immune responses and inflammatory pathology. Nat Rev Immunol.

[21] Nie S, Lu J, Wang L, Gao M (2020). Pro-inflammatory role of cell-free mitochondrial DNA in cardiovascular diseases. IUBMB Life.

[22] Rijks J, Vreugdenhil A, Dorenbos E, Karnebeek K, Joris P, Berendschot T, Mensink R, Plat J (2018). Characteristics of the retinal microvasculature in association with cardiovascular risk markers in children with overweight, obesity and morbid obesity. Sci Rep.

[23] Scarabelli T, Stephanou A, Rayment N, Pasini E, Comini L, Curello S, Ferrari R, Knight R, Latchman D (2001). Apoptosis of endothelial cells precedes myocyte cell apoptosis in ischemia/reperfusion injury. Circulation.

[24] Ji M, Cheng J, Zhang D (2022). Oxycodone protects cardiac microvascular endothelial cells against ischemia/reperfusion injury by binding to Sigma-1 receptor. Bioengineered.

[25] Querfeld U, Mak RH, Pries AR (2020). Microvascular disease in chronic kidney disease: the base of the iceberg in cardiovascular comorbidity. Clin Sci Lond Engl.

[26] Tubon I, Zannoni A, Bernardini C, Salaroli R, Bertocchi M, Mandrioli R, Vinueza D, Antognoni F, Forni M (2019). In vitro anti-inflammatory effect of Salvia sagittata ethanolic extract on primary cultures of porcine aortic endothelial cells. Oxid Med Cell Longev.

[27] Barton GM, Kagan JC (2009). A cell biological view of Toll-like receptor function: regulation through compartmentalization. Nat Rev Immunol.

[28] Fuhrmann DC, Brüne B (2017). Mitochondrial composition and function under the control of hypoxia. Redox Biol.

[29] Zhang Q, Raoof M, Chen Y, Sumi Y, Sursal T, Junger W, Brohi K, Itagaki K, Hauser CJ (2010). Circulating mitochondrial DAMPs cause inflammatory responses to injury. Nature.

[30] Bueno M, Zank D, Buendia-Roldán I, Fiedler K, Mays BG, Alvarez D, Sembrat J, Kimball B, Bullock JK, Martin JL (2019). PINK1 attenuates mtDNA release in alveolar epithelial cells and TLR9 mediated profibrotic responses. PLOS ONE.

[31] Bliksøen M, Mariero LH, Torp MK, Baysa A, Ytrehus K, Haugen F, Seljeflot I, Vaage J, Valen G, Stensløkken KO (2016). Extracellular mtDNA activates NF-κB via toll-like receptor 9 and induces cell death in cardiomyocytes. Basic Res Cardiol.

[32] Echem C, Costa TJD, Oliveira V, Giglio Colli L, Landgraf MA, Rodrigues SF, Franco M, Landgraf RG, Santos-Eichler RA, Bomfim GF (2019). Mitochondrial DNA: a new driver for sex differences in spontaneous hypertension. Pharmacol Res.

[33] Morena M, Creput C, Bouzernidj M, Rodriguez A, Chalabi L, Seigneuric B, Lauret C, Bargnoux AS, Dupuy AM, Cristol JP (2019). Randomised trial on clinical performances and biocompatibility of four high-flux hemodialyzers in two mode treatments: hemodialysis vs post dilution hemodiafiltration. Sci Rep.

